# The Impact of Benign Jawbone Tumors on the Temporomandibular Joint and Occlusion in Children: A Ten-Year Follow-Up Study

**DOI:** 10.3390/biomedicines11041210

**Published:** 2023-04-19

**Authors:** Emil Crasnean, Alina Ban, Raluca Roman, Cristian Dinu, Mihaela Băciuț, Vlad-Ionuț Nechita, Simion Bran, Florin Onișor, Teodora Badiu, Oana Almășan, Mihaela Hedeșiu

**Affiliations:** 1 Department of Maxillofacial Surgery and Implantology, Iuliu Hațieganu University of Medicine and Pharmacy, 37 Iuliu Hossu Street, 400029 Cluj-Napoca, Romania; 2 Department of Medical Informatics and Biostatistics, Iuliu Hațieganu University of Medicine and Pharmacy, 400349 Cluj-Napoca, Romania; 3 Department of Prosthetic Dentistry and Dental Materials, Iuliu Hațieganu University of Medicine and Pharmacy, 32 Clinicilor Street, 400006 Cluj-Napoca, Romania

**Keywords:** tumor, benign, odontogenic cyst, occlusion, temporomandibular joint

## Abstract

This study aimed to provide a complex analysis of the modifications in craniofacial skeleton development that may arise following the diagnosis of pediatric benign jaw tumors. A prospective study was undertaken involving 53 patients younger than 18 years of age, who presented for treatment at the Department of Maxillo-Facial Surgery, University of Medicine and Pharmacy, Cluj-Napoca, with a primary benign jaw lesion between 2012 and 2022. A total of 28 odontogenic cysts (OCs), 14 odontogenic tumors (OTs), and 11 non-OTs were identified. At follow-up, dental anomalies were identified in 26 patients, and overjet changes were found in 33 children; lateral crossbite, midline shift, and edge-to-edge bite were found in 49 cases; deep or open bite were found in 23 patients. Temporomandibular disorders (TMDs) were found in 51 children, with unilateral TMJ changes identified in 7 cases and bilateral modifications found in 44 patients. Degenerative changes in the TMJ were also diagnosed in 22 pediatric patients. Although benign lesions could be associated with dental malocclusions, a direct etiological factor could be not identified. The presence of jaw tumors or their surgical treatment could, however, be linked to a change of the occlusal relationships or the onset of a TMD.

## 1. Introduction

The jaw region is the site of numerous types of bone tumors [1]. Although pediatric patients are less affected by these lesions compared to adults, the impact of jaw tumors on children’s life is significant, since they cause alterations in facial growth and development [2].

The prevalence of pediatric jawbone tumors varies in most previous studies [2,3]. The majority of jaw tumors in children are benign [4], and according to the latest WHO classification, they are recognized [5] as odontogenic (OTs) and non-odontogenic (non-OTs), depending on their origin. Several studies have highlighted that odontoma is the most frequent OT [6]. Of all odontogenic cysts (OCs), developmental cysts, such as dentigerous cysts, are more common in children [7]. Additionally, certain non-OTs such as central giant cell tumors and aneurysmal bone cysts commonly occur within the first 20 years of life [8].

Pediatric benign jawbone tumors are often asymptomatic and are typically identified incidentally during routine dental radiographs [5]. Optimal management of these patients requires interdisciplinary work-up, complex treatment planning strategies, and post-treatment follow-up into adulthood. Treatment consists of a range of surgical procedures, including curettage, surgical excision, cryosurgery, or “en bloc” resection [9]. Of all OTs, ameloblastoma remains particularly controversial in terms of treatment, primarily due to its distinct biological behavior, characterized by slow-growth, local invasiveness, and a high recurrence rate. Compared to adult counterparts, surgeries carried out in pediatric patients are generally more conservative, as both facial growth and dental development [10] need to be evaluated.

However, the influence of these tumors on the development of the craniofacial skeleton is still poorly understood. Ameloblastomas, cemento-osseous dysplasia, fibrous dysplasia, and ossifying fibroma are examples of tumors that can enlarge the jaw and have numerous or widespread sites on the maxillary bones. Because these tumors are adjacent to important anatomical structures and developing teeth, they may result in facial abnormalities or functional limitations [11]. Other extraosseous lesions, such as tori, that develop on the lingual aspect of the jaws do not affect facial growth [12].

The growth of the craniofacial skeleton influences occlusal and jaw relationships, as well as orofacial functions [13]. Cartilaginous tissues, such as the spheno-occipital synchondrosis, nasal septal cartilage, and condylar cartilage play an important role as major growth sites for the respective anatomical structures. Among these, the condylar cartilage of the mandible is the center of greatest growth in the craniofacial complex, and it is associated with the morphogenesis of the maxillofacial complex and temporomandibular joint function [14].

In contrast to the lower jaw, the upper jaw undergoes a different growth pattern. Epiphyseal proliferation and remodeling are the two ways by which the mandible develops. Epiphyseal proliferation is the primary mechanism for bone length growth throughout the first 18 years of life. Under the condyle, the mandibular epiphysis serves as a growth site that permits the intercondylar distance to increase as the skull base widens. Mandibular remodeling occurs after growth is completed to widen the mandible [15,16].

The treatment for tumors may potentially impact a child’s mandibular growth centers. For both benign and malignant neoplasms, mandibular reconstruction with osteocutaneous free tissue transfer and titanium plate fixation has been shown to be beneficial [17].

Unlike the mandible, the maxilla does not have any endochondral growth sites, and its growth pattern is defined by an increase in vertical height and width. During maxillary growth, the maxilla is shifted inferiorly, causing remodeling along the suture lines, which promotes the development of vertical height [18].

The cranial base angle does not exert a significant influence on the emergence of dental malocclusions [19]. Numerous studies have investigated the connection between the cranial base, dental malocclusion, and jaw alignment. The findings indicate that jaw position is determined by the inclination and length of the cranial base. Abnormalities of the anterior cranial base are associated with a retrusive maxilla, while mandibular prognathism is related to various abnormalities of the posterior cranial base [20].

The development of the craniofacial skeleton is also influenced by intermaxillary occlusion. Without proper occlusion, midface and mandibular growth cessation could occur, resulting in facial asymmetry and functional alteration [21].

The presence of dental malocclusion can produce temporomandibular disorders (TMDs) [22]. TMDs commonly refer to a category of musculoskeletal conditions that affect the health of the temporomandibular joint (TMJ), masticatory muscles, and other tissues [23,24]. TMD prevalence in pediatric patients varies significantly, with estimates ranging between 4.2% and 68%, depending on the population under investigation and the assessment method employed [25,26,27]. Moreover, this prevalence appears to increase with age from childhood to adolescence [28,29]. The diagnostic criteria for TMD (DC/TMD) are based on a diagnostic protocol formulated by a group of interdisciplinary experts, including clinicians and researchers, with the goal of providing a better understanding of the diagnostics and treatment of TMD. DC/TMD protocol includes a patient’s medical history and clinical examination, imaging studies (X-rays and magnetic resonance imaging), psychological testing and blood tests. During the evaluation process, particular attention is given to symptoms associated with TMD, such as myofascial pain, difficulty eating or speaking, restricted mouth opening or closing, joint noise, or headaches [30].

To the best of our knowledge, this is the first prospective study to assess the changes in craniofacial skeleton development induced by benign jaw tumors. This research aims to provide a complex analysis of the modifications in craniofacial skeleton development, including dental malposition, dysfunctional occlusal relationships, and temporomandibular changes, that may occur following the diagnosis of pediatric benign jaw tumor.

## 2. Materials and Methods

A follow-up longitudinal cohort study was conducted at the Department of Maxillo-Facial Surgery, University of Medicine and Pharmacy, Cluj-Napoca, Romania, on pediatric patients who underwent treatment for benign tumoral lesions, over a ten-year timeframe, between January 2012 and January 2022. The study enrolled pediatric patients under the age of 18 with a histologically confirmed diagnosis of a jawbone tumor, affecting the mandible and/or the maxilla and maxillary sinus. Accessible follow-up cone beam computed tomography (CBCT) imaging (T_1_) was performed at least six months postoperatively. Patients with uncertain histopathological diagnoses, infections, soft tissue or vascular lesions, malignant jaw tumors, and salivary gland tumors and lesions were excluded from the study. Patients without CBCT images or with missing parental consent for clinical examinations or additional investigations, including follow-up, were excluded from this study. Additionally, patients with limited CBCT field of view images were excluded due to limitations in establishing a diagnosis.

The preoperative and postoperative CBCT scans were obtained using the same equipment and imaging protocol (Promax 3D Max, Planmeca, Finland). The following CBCT scan parameters were analyzed by two experienced radiologists (M.H. and R.R.): dental anomalies (tooth malposition or impaction), malocclusion (jaw relationship in the sagittal, transversal and vertical planes, inter-canine, inter-first premolar, and inter-first molar distances), temporomandibular joint condyle position, and bone morphology changes.

Inter-canine, inter-first premolar, and inter-first molar widths were measured on the preoperative and postoperative CBCT coronal images in both maxillary and mandibular jaws. Inter-canine width was measured from the cusp tips of the right and left canine. The inter-first molar width was determined as the distance between the mesiobuccal cusp tips of the right and left first permanent molars. The inter-first premolar width was measured as the distance between the tips of the buccal cusps.

The condylar position (anterior, posterior, or centric) was assessed using oblique sagittal and coronal reformatted CBCT images, according to the Pullinger et al. method [31].

Inter-rater reliability for all measurements was evaluated by two experienced independent examiners. Intra-rater reliability was assessed by conducting two separate measurements performed by the primary investigator (for the first 15 participants) at a two-week interval.

The study was approved by the Ethical Committee of the University of Medicine and Pharmacy ‘Iuliu Hațieganu’, Cluj-Napoca, Romania, DEP 227 (5 July 2022).

### Statistical Methods

The statistical analysis was performed using the R Commander software (R Foundation for Statistical Computing, Vienna, Austria) version 4.0.5. Quantitative data distribution was assessed using the Shapiro–Wilk test, skewness, and kurtosis values. For normally distributed data, results were presented as mean and standard deviation, whereas for non-normal distribution, the median and interquartile range (IQR) were used. Comparison of quantitative data was obtained using the Wilcoxon test for pre-postoperative evaluation. For normally distributed data, the Student t-test was employed. For qualitative data, the results were presented as absolute and relative frequencies. Frequencies were compared through the Stuart–Maxwell Marginal Homogeneity Test with Monte Carlo resampling approximation.

Results were considered statistically significant if the *p*-value was lower than 0.05. The intra-and inter-rater reliability data were analyzed using the two-way random effect model and were expressed using the intra-class correlation coefficient (ICC) and its corresponding 95% confidence interval (CI).

## 3. Results

### 3.1. General Follow-Up Data

The total sample included 53 pediatric patients (29 males and 24 females) who underwent CBCT imagistic follow-up. Imaging follow-up was carried out between 6 and 118 months postoperatively (radical excision, marsupialization, biopsy, or reconstruction). Twenty-five patients underwent a preoperative CBCT examination (T_0_). Other cases (28 patients) underwent different radiological investigations assessing their preoperative status.

The mean age of the follow-up pediatric patients was 15.1 ± 4.1 (with an age range from 4 years to 22 years). A total of 28 odontogenic cysts (OCs), 14 odontogenic tumors (OTs), and 11 non-OTs were identified at follow-up imaging (Table 1).

The mandible was the most common location for the tumors (64.1%). The most frequent surgical procedure performed was tumor enucleation (84.9%), followed by jaw reconstruction in 0.9% of cases. Simple biopsy was performed in 0.7 % of the patients, while marsupialization was performed only in 4 cases (0.7%).

The median time for the CBCT follow-up examination was 49.8 ± 29.2 months post-operatively. During the follow-up period, recurrence was observed only in one case of odontogenic keratocysts (1.8% of all pediatric jaw tumors) (Table 2).

### 3.2. Dental Anomalies and Jaw Relationship

At follow-up, from a total number of 53 patients, 27 patients did not exhibit any dental anomalies (50.1%). Dental relationships were found to be normal in the sagittal plane (20%), in the transversal plane (28.3%), and in the vertical plane (56.6%).

Overall, the results showed that a total of 26 pediatric patients had at least one dentoalveolar development anomaly. Dental anomalies were identified in 26 cases (49%, tooth malposition in 21 cases, impacted teeth in 5 cases); overjet changes were found in 33 patients; a total of 49 cases exhibited lateral crossbite, midline shift, and edge-to-edge bite; deep or open bite was found in 23 patients (Table 3).

### 3.3. Temporomandibular Joint

The centric position of the condyle was found in 65 temporomandibular joints (61.3%). Temporomandibular disorders were noted in 51 (96.2%) patients; unilateral TMJ changes were identified in 7 cases; and bilateral modification was found in 44 patients. The most frequent TMJ pathology was condyle flattening (57.5%). Degenerative changes in the temporomandibular joint were also diagnosed in 22 (20.7%) pediatric patients (Table 4).

### 3.4. CBCT Comparison in the Preoperative and Postoperative Status

From a total number of 53 patients, only 25 children and adolescents were assessed using preoperative and postoperative CBCT. A comparison between preoperative and postoperative dentoalveolar anomalies and TMJ is summarized in Table 5.

### 3.5. CBCT Dental Measurements

Comparison of CBCT dental measurements between T_0_ and T_1_ revealed no statistical significance (Table 6).

We also found that all intra- and inter-rater reliabilities for measurements were greater than 0.8, which is considered excellent according to Cicchetti’s classification [32].

## 4. Discussion

The overall prevalence of the reported pediatric bone tumors varies widely depending on the type of tumoral classification applied. Our findings suggest overall male dominance and a higher incidence of mandibular cases, which is consistent with previous studies [2,33,34]. The current investigation also revealed that a majority of inflammatory pediatric jaw cysts were odontogenic tumors (OT) (52.8%), contrasting with the results identified by other studies [35].

Occurrence of lesions and tumors was most frequently observed among patients in their second decade of life. Jaw tumor development is also considered to occur predominantly within the second decade of a child’s life [36,37,38]. The findings of this study confirm this hypothesis. This may be explained by the transitions from mixed to permanent dentition, and it is worth highlighting that the greatest proportion of the follow-up patients included in our study were adolescents (15.1 ± 4.1 years old) (Table 2).

Cancer diagnosis in children and adolescents can result in dental anomalies and disorders ranging from mild to severe [39]. Hypodontia, microdontia, enamel defect, and root malformation are the most common dental anomalies found in cancer survivors [40]. Our study highlighted the prevalence of dental malposition and impacted teeth (49%) (Table 3). These results indicate that different types of anomalies can be observed, contingent on whether the jaw tumor is malignant or benign.

In our research, it was noted that more than half of the patients (64%) presented changes in tooth position during the preoperative period, while 28% suffered from impacted teeth due to the presence of the tumor. Following surgical intervention, most of the patients received orthodontic treatment that corrected most of the dental malposition. Our research shows that dental malposition could arise in pediatric patients who did not receive orthodontic treatment following the surgical procedure (40% of patients), emphasizing the importance of an interdisciplinary approach to pediatric jawbone tumors and lesions. The predominant surgical option employed for children with impacted teeth was radical tumor excision with tooth extraction. Overall, careful consideration must be given to the surgical treatment for impacted teeth in pediatric patients to avoid potential disturbances in dental eruption and the dental alignment of permanent dentition.

Malocclusion is one of the most important dental modifications, with prevalence estimates ranging from 20% to 100%, according to various studies [41,42,43]. Midline deviation, deep overbite, increased overjet, and crossbite are frequently found in children and adolescents (36), and the present research reveals a comparable pattern of results. The majority of patients (72%) presented with an increased overjet during the preoperative period. Midline shift (13 patients) and edge-to-edge bite (9 patients) were the most common modifications found in the transversal plane. However, open bite was the most consistent change in the vertical plane (52%). These findings suggest that the majority of patients exhibited malocclusion in the preoperative period. Furthermore, these results demonstrate that jawbone lesions or tumors have the potential to induce or maintain dental malocclusion.

Conversely, various other factors may be associated with occlusal disorders during the preoperative period. Ectopic eruption or dental malposition could be regarded as important factors in the development of malocclusions prior to surgical treatment. Dental caries or dental pain could produce unilateral mastication, altering the distribution of occlusal forces. In addition, trauma of the primary teeth, periapical lesions of the deciduous teeth, abnormal tooth development, or different oral habits could also be key factors that contribute to dental malocclusion [44]. Our study revealed that the majority of dental occlusion modifications were corrected or improved after surgical treatment via orthodontic therapy.

On the other hand, in some cases, the emergence of new malocclusion was noted. In preoperative status, none of the patients had crossbite or scissor-bite modification. Following surgical treatment, changes in the transverse plane were identified in three patients (12%). This could be attributed to either the absence of orthodontic treatment or to a particular type of surgical treatment. Posterior crossbite is considered to be the most frequent dental malocclusion in primary and mixed dentition, occurring in 8% to 22% of the cases [45]. The main cause of postoperative crossbite could be the reduction in the width of the maxillary arch after surgical treatment. Additionally, it is worth noting that 13 patients presented with midline shifts at the preoperative evaluation, while 2 additional patients showed mandibular deviation at follow-up. This postoperative occlusal modification (mandibular deviation and crossbite) is reported to produce changes in the size of the jaws and occlusal interference according to some studies [46]. At the same time, midline shift and/or posterior crossbite have been found to cause temporomandibular dysfunction, potentially leading to disturbance of facial growth in children.

Our results cast new light on the importance of dental occlusal analysis following surgical treatment in identifying and preventing future complications. Drawing on our expertise, we contend that surgical procedures may induce dental malocclusion, especially in young patients who have not received orthodontic treatment. However, the main limitation of our research is the relatively small sample size of patients (n = 25) who underwent preoperative and postoperative imaging. To overcome this limitation and extend the generalizability of our findings, a multicenter collaboration study would be recommended.

Tumor size in our pediatric cohort ranged from 1 cm to 8 cm. This large variation may suggest that most occlusal alterations were not directly attributable to the presence of the tumor, but rather they aggravated an already existing malocclusion.

Serving as one of the growth centers of the jaw, the TMJ condylar cartilage has the capacity to adapt to the physiological changes of the occlusion. Hence, occlusal stress, trauma, the presence of tumors, and malocclusions can induce abnormal mechanical stress to the TMJ, ultimately contributing to degenerative changes and remodeling of the joint. TMJ osseous degenerative changes include sclerosis, erosion, condyle flattening, osteophyte, subchondral cysts, and narrowing of the joint space [47,48]. In our study, patients with malocclusions presented TMJ degenerative changes (Table 5). However, new cases of TMD (86%) were also identified postoperatively. Occlusal instability with posterior crossbite has been observed in 12% of our patients, although different results were obtained by Krasteva et al. [46]. Postoperatively, condyle flattening (86%) and degenerative changes (32%) were found in patients who did not have TMJ alterations before the surgical intervention. Several studies suggested that distally positioned condyles could predict the development of TMD [49]. However, in our study, only seven children exhibited posterior condyle position in preoperative status, and only one case was corrected after surgical treatment. Therefore, our results show that distal condyle positioning does not significantly impact the occurrence of TMD.

Our findings also suggest that surgical treatment of benign jaw tumors and lesions does not produce skeletal changes or transverse bimaxillary deficiency. Additionally, no statistically significant difference between maxillary and mandibular dental measurements were found at different follow-up periods (T_0_–T_1_) (Table 6). The treatment of small benign tumors usually involves a minimally invasive approach and does not require complex bone reconstruction [50]. However, in cases where surgical resection is required, it is mandatory to preserve the condylar and the subcondylar growth center [51], as several studies have shown that extensive or radical surgical treatment can result in developmental disorders of the jaws [21,52]. In our study, the mean size of the tumoral lesions was 2.9 ± 1.4 cm. Nevertheless, a higher number of patients with large jawbone tumors is needed to establish the possibility of transversal jaw deficiency. Therefore, a multicenter collaboration would be desirable to corroborate our results.

The use of radiotherapy and chemotherapy as adjuvant therapies in pediatric malignancies and benign tumors is rarely required. However, in certain histopathological forms such as ameloblastoma, adjuvant radiotherapy/chemotherapy may be necessary [53]. Radiotherapy can induce alterations in dental eruption, and it has been demonstrated that a dose of 10 Gy could generate irreversible changes in ameloblasts, while a dose of 30 Gy could stop dental development [54]. Animal studies have shown that chemotherapy can also produce severe dental developmental disorders [55,56]. While this type of alterations were not observed in our study, it is extremely important to identify the possible dental alterations resulting from these therapies.

When dealing with this variety of tumors and lesions in the pediatric population, it is crucial to promptly identify the signs and symptoms of a tumor, perform pre- and post-surgical imaging evaluations, and assess the dental occlusion and TMJ status both preoperatively and postoperatively. A multidisciplinary approach, including orthodontic therapy and surgical treatment, may contribute to a favorable follow-up of the occlusal changes and of the TMJ status.

## 5. Conclusions

Pediatric jaw tumors and lesions are rare, and the epidemiology, clinical characteristics, radiographic findings, and treatment principles of pediatric jaw tumors differ from those of adults. Our study revealed a significant prevalence of dental malposition and impacted teeth among pediatric patients with jawbone tumors and lesions. It was observed that the majority of children exhibited malocclusion at the preoperative stage. Our study has revealed that jawbone lesions or tumors could induce or aggravate dental malocclusion. We also concluded that surgical procedures might result in dental malocclusion, particularly in young patients who have not received orthodontic treatment. The occurrence of a jaw tumor or its surgical treatment may be associated to alterations of the occlusal relationships or the onset of a temporomandibular disorder. Our research demonstrated a significant correlation between malocclusions and TMJ degenerative changes in our patient cohort. Further investigations involving a larger sample size are required to establish the relationship between bimaxillary transversal deficiency and benign pediatric jawbone lesions or tumors.

## Figures and Tables

**Table 1:** Distribution and prevalence of follow-up pediatric jaw lesions (N = 53).

Jaw Lesion	N (%) *
**Benign odontogenic tumors (OTs)**	14 (26.4)
Epithelial	
Ameloblastoma	2 (3.7)
Mesenchymal	
Odontogenic fibroma	1 (1.8)
Odontogenic myxoma	5 (9.4)
Mixed	
Ameloblastic fibroma	1 (1.8)
Odontoma	5 (9.4)
**Benign nonodontogenic tumors (non-OTs)**	11 (20.7)
Maxillofacial bone tumors	
Osteoma	1 (1.8)
Osteoid osteoma	1 (1.8)
Desmoplastic fibroma	1 (1.8)
Fibro-osseous tumors	
Fibrous dysplasia	3 (5.6)
Giant cell lesions and bone cysts	
Giant cell granuloma	3 (5.6)
Simple bone cyst	1 (1.8)
Cherubism	1 (1.8)
**Odontogenic cysts (OCs)**	28 (52.8)
Inflammatory	
Radicular cyst	13 (24.5)
Developmental	
Dentigerous cyst	6 (11.3)
Odontogenic keratocysts	9 (16.9)

N—number of jaw lesions; * absolute values and percentages.

**Table 2:** General follow-up data findings for pediatric patients with jaw tumors and lesions (N = 53).

	Total N (%)	Odontogenic Tumors (OTs) N (%)	Non-Odontogenic Tumors (Non-OTs) N (%)	Odontogenic Cysts (OCs) N (%)
**Gender**				
Male	29 (54.7)	7 (50)	6 (54.5)	16 (57.1)
Female	24 (45.2)	7 (50)	5 (45.4)	12 (42.8)
**Size (cm)**	2.9 ± 1.4	2.8 ± 0.9	3.9 ± 2	2.6 ± 1.3
**Age**	15.1 ± 4.1	11.7 ± 4.7	15.2 ± 2.2	16.8 ± 3.2
**Tumor location**				
Mandible	34 (64.1)	8 (57.1)	6 (54.5)	20 (71.4)
Maxillary	19 (35.8)	6 (42.8)	5 (45.4)	8 (28.5)
**Treatment**				
Simple biopsy	4 (0.7)	0 (0)	4 (36.3)	0 (0)
Excision	45 (84.9)	13 (92.8)	7 (63.6.5)	25 (89.2)
Reconstruction	5 (0.9)	3 (21.4)	1 (0.9)	1 (0.3)
Marsupialization	4 (0.7)	1 (0.7)	0 (0)	3 (10.7)
**Follow-up**				
Median	49.8 ± 29.2	41.7 ± 28.7	70.2 ± 29.8	45.8 ± 26.3
Range	6–118	7–100	33–118	6–118
**Recurrence**	1 (1.8)	0 (0)	0 (0)	1 (1.8)

Data presented as N (%) and median ± standard deviation for follow-up (months), respectively, for the follow-up age of the children.

**Table 3:** Dental anomalies and jaw relationship in imagistic follow-up for pediatric patients with jaw tumor (N = 53).

	Total N (%) *	Odontogenic Tumors (OTs) N (%) *	Odontogenic Cysts (OCs) N (%) *	Non-Odontogenic Tumors (Non-OTs) N (%) *
**Dental anomalies**				
Malposition	21 (39.6)	10 (71.4)	8 (28.5)	3 (27.2)
Impacted teeth	5 (9.4)	1 (7.1)	2 (7.1)	2 (18.1)
**Malocclusion**				
**Sagittal plane**				
Normal sagittal	20 (37.7)	7(13.2)	12 (42.8)	1 (9)
Increased Overjet	31 (58.4)	7 (50)	16 (57.1)	8 (72.7)
Negative Overjet	2 (3.7)	0 (0)	2 (7.1)	0 (0)
**Transverse plane**				
Normal transverse	15 (28.3)	4 (28.5)	9 (32.1)	2 (18.1)
Cross bite, scissor bite	8 (15)	2 (14.2)	4 (14.2)	2 (18.1)
Midline shift	30 (56.6)	7 (50)	15 (53.5)	8 (72.7)
Edge-to-Edge bite	11 (20.7)	3 (21.4)	7 (25)	1 (9)
**Vertical plane**				
*Normal vertical*	30 (56.6)	10 (71.4)	15 (53.5)	5 (45.4)
Deep bite	7 (13.2)	3 (21.4)	3 (10.7)	1 (9)
Open bite	16 (30.1)	3 (21.4)	5 (17.8)	8 (72.7)

N = number of patients; * absolute values and percentages; OTs—odontogenic tumors; non-OTs—nonodontogenic tumors; OCs—odontogenic cysts.

**Table 4:** Temporomandibular joint changes in follow-up imaging found in pediatric patients with jaw tumors (N = 106).

TMJ Changes	Total N (%) *	Odontogenic Tumors (OTs) N (%) *	Odontogenic Cysts (OCs) N (%) *	Non-Odontogenic Tumors (Non-OTS) N (%) *
Normal condyle position	65 (61.3)	18 (64.2)	35 (62.5)	12 (54.5)
Anterior condyle position	21 (19.8)	6 (21.4)	10 (17.8)	5 (22.7)
Posterior condyle position	15 (14.1)	4 (14.2)	9 (16)	2 (9)
Superior condyle position	5 (4.7)	0 (0)	2 (3.5)	3 (13.6)
Medial position	36 (33.9)	13 (46.4)	14 (25)	9 (40.1)
Lateral position	24 (22.6)	5 (17.8)	14 (25)	5 (22.7)
Condyle flattening	61 (57.5)	18 (64.2)	28 (50)	15 (68.1)
Degenerative bone changes	22 (20.7)	5 (17.8)	14 (25)	3 (13.6)

N = total number of TMJ; * absolute values and percentages; OTs—odontogenic tumors; non-OTs—nonodontogenic tumors; OCs—odontogenic cysts.

**Table 5:** Comparison of the preoperative and postoperative dentoalveolar anomalies and TMJ changes on CBCT examination associated with pediatric bone tumors (N = 25).

	Preoperative T_0_ N(%) *	Postoperative T_1_ N(%) *	*p*-Value (T_0_, T_1_)
**Dental anomalies**			
Malposition	16 (64)	10 (40)	0.06
Impacted teeth	7 (28)	0 (0)	0.16
Malocclusion			
**Sagittal plane**			
Normal Overjet	5 (20)	7 (28)	0.62
Increased Overjet	18 (72)	16 (64)	0.62
Negative Overjet	2 (8)	2(8)	0.99
**Transversal plane**			
Normal transversal	3 (12)	0 (0)	0.25
Cross bite, scissor bite	0 (0)	3 (12)	0.25
Edge-to-edge bite	9 (36)	7 (28)	0.67
Midline shift	13 (52)	15 (60)	0.50
**Vertical plane**			
Normal Vertical	6 (24)	14 (56)	0.07
Deep bite	6 (24)	5 (20)	0.99
Open bite	13 (52)	6 (24)	0.11
TMJ changes **			
**Sagittal plane**			
Normal condyle position	29 (58)	32 (64)	0.58
Anterior condyle position	13 (26)	12 (24)	0.99
Posterior condyle position	7 (14)	6 (12)	0.99
Superior condyle position	1 (2)	0 (0)	0.99
**Coronal plane**			
Central position	21(42)	18(36)	0.59
Medial position	20 (40)	18 (36)	0.72
Lateral position	9 (18)	14 (28)	0.12
**Condyle flattening**	42 (84)	43 (86)	0.67
**Degenerative bone changes**	14 (28)	16 (32)	0.99

N—number of patients; * absolute values and percentages; **—50 TMJs from 25 patients.

**Table 6:** Inter-canine, inter-first premolar, and inter-first molar CBCT measurements in preoperative and postoperative assessments (N = 25).

	Preoperative (T_0_) *	Postoperative (T_1_) *	*p*-Value
**Upper jaw**			
Inter-canine distance	35.5 ± 3.9	36.3 ± 3	0.07
Inter-first premolar	44.1 [39.2–44.9]	43.8 [40.6–44.4]	0.38
Inter-first molar	52.3 ± 4.1	53.2 ± 3.3	0.06
**Lower jaw**			
Inter-canine distance	28.9 [27.6–30.3]	29.2 [20.1–30.1]	0.36
Inter-first premolar	38.5 ± 3.1	38.8 ± 2.3	0.89
Inter-first molar	50.6 ± 3.1	50.9 ± 3	0.39

* Distance measured in millimeters (mm). For data with normal distribution, results were presented as average and standard deviation; for asymmetric distribution, results were presented as median and interquartile range.

## Data Availability

The data presented in this study are available on request from the corresponding author. The data are not publicly available due to due to restrictions: privacy and ethical.

## Abbreviations

Temporomandibular joint	TMJ
Odontogenic Cysts	OCs
Odontogenic Tumors	OTs
Non-odontogenic Tumors	non-OTs
Temporomandibular disorders	TMDs
Diagnostic Criteria for temporomandibular disorders	DC/TMD
Cone Beam Computed Tomography	CBCT
